# Rhythms of human attention and memory: An embedded process perspective

**DOI:** 10.3389/fnhum.2022.905837

**Published:** 2022-10-05

**Authors:** Moritz Köster, Thomas Gruber

**Affiliations:** ^1^Faculty of Education and Psychology, Freie Universität Berlin, Berlin, Germany; ^2^Institute of Psychology, University of Regensburg, Regensburg, Germany; ^3^Institute of Psychology, Osnabrück University, Osnabrück, Germany

**Keywords:** brain rhythms, embedded-process model, theta-gamma coupling, alpha oscillations, human cognition

## Abstract

It remains a dogma in cognitive neuroscience to separate human attention and memory into distinct modules and processes. Here we propose that brain rhythms reflect the embedded nature of these processes in the human brain, as evident from their shared neural signatures: gamma oscillations (30–90 Hz) reflect sensory information processing and activated neural representations (memory items). The theta rhythm (3–8 Hz) is a pacemaker of explicit control processes (central executive), structuring neural information processing, bit by bit, as reflected in the theta-gamma code. By representing memory items in a sequential and time-compressed manner the theta-gamma code is hypothesized to solve key problems of neural computation: (1) attentional sampling (integrating and segregating information processing), (2) mnemonic updating (implementing Hebbian learning), and (3) predictive coding (advancing information processing ahead of the real time to guide behavior). In this framework, reduced alpha oscillations (8–14 Hz) reflect activated semantic networks, involved in both explicit and implicit mnemonic processes. Linking recent theoretical accounts and empirical insights on neural rhythms to the embedded-process model advances our understanding of the integrated nature of attention and memory – as the bedrock of human cognition.

## Introduction

“Everything in life is memory, save for the thin edge of the present.” (Gazzaniga, 2000).

Yet also our present, at least everything we perceive of it, is a mnemonic process. When awake, the human brain constantly samples, interprets, and elaborates novel information from the environment, based on former experience, to integrate them into a coherent representation of the world around (Buzsáki, 1996). At the same time, the interpretation and integration of sensory information forms the basis for predictions about future events and behavioral navigation (i.e., active inference), but also for vividly re-experiencing episodes later in life. In this sense, we construe memory as an integral function of human cognition, from re-experiencing the past and making sense of the present, to guiding future behavior.

Human memory relies on associative neural networks (Anderson and Bower, 1973; Tulving and Craik, 2000), which are shaped by experience, from orientation cells in the primary visual cortex (Blakemore and Cooper, 1970), to highly specialized expert and decision networks (Gauthier et al., 2000; Rushworth et al., 2011). This points to the key question about the neural dynamics, which allow the brain to process novel sensory inputs, to interpreted them in the context of existing associations and to integrate them by changing the structure of these networks.

Complementing our understanding from psychology (e.g., Cowan, 1997) and the brain structures involved in attention and memory (e.g., Squire and Wixted, 2011), there is a growing consensus that information processing in the human brain is rhythmic (e.g., VanRullen, 2016; Fiebelkorn and Kastner, 2019). Rhythmic brain activity is thought to integrate and organize neural processes in humans and other mammalian species (Buzsáki, 1996; Engel et al., 2001; Varela et al., 2001; Fell and Axmacher, 2011; Fries, 2015). While the debate on the functional role of neural rhythms continues (i.e., rather than being epiphenomenal, cf. Buzsaki, 2004; e.g., arising from network properties, e.g., Lundqvist et al., 2011; Kovach et al., 2018), one critical argument for the functional role of rhythmic brain activity are the recent advances made by neural stimulation methods, demonstrating that attention and memory processes can be experimentally manipulated by externally applied rhythms (Alekseichuk et al., 2016; Clouter et al., 2017; see Hanslmayr et al., 2019, for a review; Köster et al., 2019b; Reinhart and Nguyen, 2019; Albouy et al., 2022). Thus, we will here build on the assumption that neural rhythms indeed reflect key mechanisms in implementing human cognitive functions (see, Obleser and Kayser, 2019, for critical considerations on neural entrainment).

In this perspective article, we synthesize the state-of-the-art on neural dynamics in human attention and memory processing and propose how they may realize the elements proposed of the embedded process model (Cowan, 1997), derived from human psychology, on the neural architecture of the human brain. Specifically, experimental psychologists have overcome isolated perspectives on human attention and memory, such as attentional selection (Posner and Petersen, 1990) working memory (Baddeley, 1978), and memory formation (Hebb, 1949). Cowan’s embedded-processes model (Cowan, 1997) characterizes how attention and memory act in concert in everyday cognition to guide human behavior. Given its wide appreciation, this model provides an excellent basis for a theoretical integration.

In the framework offered here, we sketch how neural rhythms may realize the elements of the embedded process model on the neural architecture of the human brain, providing a theoretical basis for a unified understanding of human attention and memory, in psychology and cognitive neuroscience. First, we characterize Cowan’s embedded process model and the brain regions involved in human memory. We will then highlight, how rhythmic neural dynamics may reflect the specific elements of the Cowan model, drawing from prevailing accounts on neural rhythms of human attention and memory. To exemplify our idea, we propose the concept of an attentional mnemonic updating loop, implemented by the theta-gamma neural code (cf. Lisman and Jensen, 2013).

## Attention and memory as embedded processes

**FIGURE 1:**
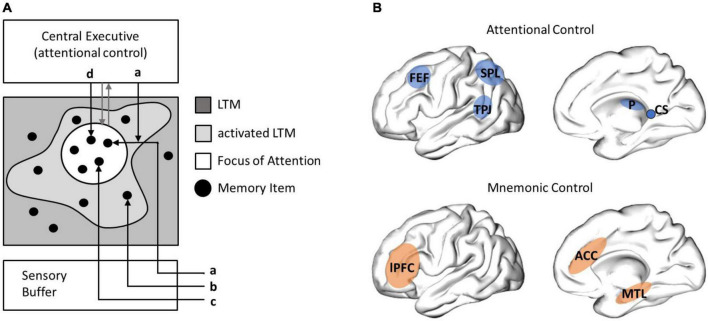
Cowan’s embedded-process-model and neuroanatomical structures of attentional and mnemonic control. **(A)** Cowan (1988) suggests that attention and memory processes act on activated representations in long-term memory (LTM, so called memory items). The focus of attention is controlled by the central executive (gray arrows) and lies on a subset of activated items: sensory information may be (a) selectively attended, (b) guide automated behavior, if they are habituated, (c) enter the focus of attention immediately, if unexpected and thus dishabituated, or (d) activated by central executive processes. Cowan emphasizes that information processing serves behavioral navigation, namely controlled actions, guided by the central executive, or automated actions, based on highly habituated processes (not illustrated in this graph). **(B)** A central role for selective attention processes is ascribed to the frontal eye field (FEF) and the superior colliculi (CS), in eye-movement control, as well as the pulvinar (P), the superior parietal lobe (SPL) and the temporal parietal junction (TPJ), in cortical attentional control (adapted from Posner and Rothbart, 2007). A central role for mnemonic control processes has been ascribed to the lateral prefrontal cortex (lPFC), the anterior cingulate cortex (ACC), and the medial temporal lobe (MTL).

When awake, the human brain continuously samples novel information, interprets this information based on former experience, and integrates it into existing neural networks, to maintain a coherent representation of time and space (Eichenbaum, 2017; Buzsáki and Tingley, 2018). Psychological models propose that attention and memory processes are inextricably linked to each other (Craik and Lockhart, 1972; Cowan, 1988). A model that specifies the integrated nature of human information processing and memory is the embedded-process-model (Cowan, 1988; Figure 1A). In this model explicit memory processes are directed by a central executive. The central executive sets the focus of attention, which operates on activated representations in long-term memory (LTM). Representations in LTM may be activated by sensory information but may also be activated internally by the attentional focus of the central executive (see routes *a–d*, Figure 1A). Cowan proposes a sensory buffer for all incoming sensory information, which automatically activates representations in LTM. This information may decay rapidly, get into the focus of attention, to be involved in explicit memory processes, or be involved in implicit processes, without getting into the focus of attention and being mentally elaborated. This accounts for automated information processing and behavior (see route *b*, Figure 1A).

Cowan (1988) characterizes representations in LTM as memory items. A memory item represents a cluster of highly interconnected nodes (i.e., cell assemblies) within associative neural networks. This principal idea is based on chunking phenomena (e.g., the letters F B I may form a single memory item; Cowan, 2001). To add a critical aspect to Cowan’s basic concept of a memory item, neural representations do not merely serve a static internal representation of the environment, but ultimately serve behavioral navigation (O’Regan and Noë, 2001; Engel et al., 2013). Thus, a neural representation (e.g., of a cup) does not only include perceptual features, but also the motor programs concerning how to grasp it or how to refer to it in a conversation.

Cowan’s model has received much attention, because it is compatible with numerous findings from experimental psychology, including chunking processes and working memory capacity (Cowan, 2001), perceptual priming (Tulving and Schacter, 1987), and the depth of processing account (Craik and Lockhart, 1972). Importantly, the model provides a plausible framework of how attention and memory work together, on one and the same neural architecture. At the same time, it provides a theoretical separation between explicit and implicit processes. That is, memory items which are in the present focus of attention are explicitly processed by the central executive (i.e., elaborated in working memory) and may thus be explicitly remembered later on (declarative memory), while memory items may also be involved in implicit processes and automated behavior (non-declarative memory).

In the following, we first revisit critical brain structures involved in human memory. Then, based on the elements specified in the Cowan model, we elaborate how neural oscillations may link attention and memory processes to the neural architecture of the human brain.

Of note, Cowan already suggested that neural rhythmic activity (40 Hz gamma oscillations) may play a critical role in implementing the elements of the model (Cowan, 1997) and specified his model with the underlying neuroanatomy in mind (Cowan, 2019). However, there is no comprehensive framework, to date, that integrates the Cowan’s model and relevant brain structures with neural oscillatory dynamics involved in human attention and memory.

## Brain structures of attention and memory

Perceptual information is processed in primary sensory regions and fed forward into associative networks. This is well characterized for the visual system (Felleman and Van Essen, 1991; Bosman et al., 2012): Along the hierarchy of the visual cortex, perceptual information converges on increasingly abstract representations, for example in the inferior temporal (IT) cortex (Tanaka, 1993), and is complemented by spatial information in parietal networks (Goodale and Milner, 1992), to be integrated into objects and scenes (Corbetta et al., 1995).

The semantic information of different modalities (visual, auditory, and tactile) is represented in widely distributed associative networks in the neocortex (NC) and the medial temporal lobe (MTL; Binder and Desai, 2011). The MTL is, at the same time, ascribed a key role in memory formation (e.g., Scoville and Milner, 1957; Kim and Fanselow, 1992; Squire and Alvarez, 1995). Specifically, the MTL may act as an intermediate storage system, where novel associations are formed rapidly before they are transferred into lasting semantic networks in the NC (dual memory systems account; Squire and Alvarez, 1995). This avoids interferences between fast updating contextual information (in the MTL) and long-lasting semantic information (in the NC; Halgren, 1988; McClelland et al., 1995). Neurons in MTL networks represent sparse and abstract information about the external world (Buzsáki, 1996). For example, place cells (O’Keefe and Recce, 1993), and cells in the MTL that encode specific people, objects (Quiroga et al., 2005), or places (Jacobs et al., 2013). As an intermediate storage system, the MTL is assigned a critical role in storing contextual information about current time and space (Eichenbaum, 2017; Buzsáki and Tingley, 2018). This (neurophysiologically inspired) dual process-account adds to the Cowan model the division of labor between NC and MTL networks in human memory (see also, Cowan, 2019).

Control networks (see Figure 1B) serve the dynamic activation and integration of distributed neural processes into a functional mnemonic architecture that fulfills current task demands. Mnemonic control strongly relies on the prefrontal cortical networks (PFC; Brewer, 1998; Wagner, 1998; Kirchhoff et al., 2000), with the PFC being ascribed an executive control function (Blumenfeld and Ranganath, 2007): The lateral PFC is involved in the selection and ordering of multiple pieces of information, for example during WM maintenance. The anterior cingulate cortex (ACC), as part of the PFC control network, plays a key role in experience-based predictions (Jahn et al., 2014). Furthermore, the control systems for selective (top-down) attention include thalamic regions, such as the pulvinar, and tempo-parietal regions, as well as the frontal eye-fields and the superior colliculi, which directly control attention processes by directing eye-movements (see Figure 1B; for a review, see Posner and Rothbart, 2007).

Linking those neural structures to the elements of the Cowan model, central executive processes may be implemented in the PFC, in concert with further attention and memory control networks (Figure 1A). These executive processes may act on activated semantic representations in the NC (Binder and Desai, 2011), and information on the current time and space in MTL networks (Miller et al., 2013).

We will now turn to the critical question of how attentional and mnemonic processes are orchestrated temporally. Namely, how neural oscillatory dynamics may reflect the basic elements of the Cowan model and realize them on the neural architecture of the human brain.

## Neural rhythms – temporal dynamics of neural computation

It is a key question, how distributed brain processes are integrated and bound into coherent experiences (e.g., in object perception; Treisman and Gelade, 1980). Initially discovered in the cat visual cortex (Gray et al., 1989), by now, there is profound empirical evidence that the integration and organization of distributed brain processes is rhythmic (e.g., Fries, 2015), and that rhythmic network dynamics facilitate memory processes (e.g., Fell and Axmacher, 2011). Rhythmic brain activity is phylogenetically preserved in the mammalian lineage (Buzsaki, 2004) and has been well characterized at the microscopic (e.g., Fell and Axmacher, 2011), the mesoscopic (e.g., Fries, 2015), and the macroscopic level (e.g., Roux and Uhlhaas, 2014; Hanslmayr et al., 2016). In the following, we first introduce the basic neural mechanisms which have been associated with gamma, theta and alpha oscillations. Thereafter, we discuss their potential role in human attention and memory, by linking empirical findings from cognitive neuroscience to the elements of the Cowan model.

### Gamma oscillations

**FIGURE 2:**
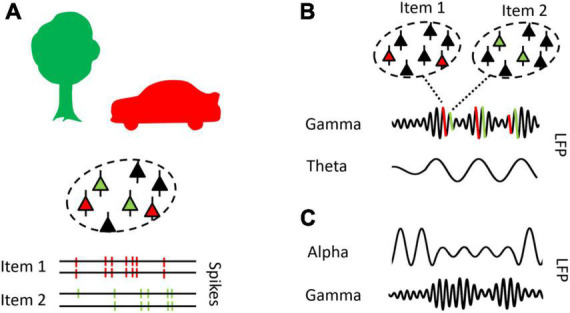
Theta, alpha, and gamma oscillations reflect distinct neural mechanisms. **(A)** Neurons which encode the same object (i.e., item) synchronize firing activity within and across cell assemblies in a gamma pace (>30 Hz) (cf., Gray et al., 1989). **(B)** The theta rhythm (3–8 Hz) is presumed to organize neural processes, as reflected in a theta-gamma neural code, a coding schema proposed to serve the segregation and ordering of neural representations (cf., Lisman and Jensen, 2013). **(C)** Alpha (8–14 Hz) oscillations by act as a gating mechanism, inhibiting neural processes in task irrelevant networks and facilitate information processing in task relevant networks, such that reduced alpha power coincides with increased gamma power (cf., Jensen and Mazaheri, 2010).

In their seminal work, Gray et al. (1989) found that neurons in the cat visual cortex synchronize their activation pattern in the gamma frequency (>30 Hz) when they encode features of the same object. For example, this may be the case for neurons that encode the shape and the color of a red car (see Figure 2A). According to the binding by synchrony theorem, perceptual information, encoded by neurons distributed across the brain, is integrated by synchronized firing activity (Engel and Singer, 2001). Specifically, the coherence of neural firing in the gamma frequency has been shown to promote visual perceptual information along the visual cortical hierarchy (Bosman et al., 2012) and to synchronize neural activity within (Gray et al., 1989) and across (Womelsdorf et al., 2007) cell assemblies (see Fries, 2015, for a review). On the cellular level, gamma oscillations may promote and sustain activity in neural networks based on inhibitory interneuron (ING) or inhibitory as well as excitatory pyramidal-interneuron (PING) GABAergic activity (e.g., Tiesinga and Sejnowski, 2009; Buzsáki and Wang, 2012; for computational models, see, Mazzoni et al., 2015; Keeley et al., 2017).

Conceptually, it has been proposed that early gamma activity reflects the activation of existing representations by sensory information, which are then further utilized by higher cognitive processes, reflected in later gamma bursts (Herrmann et al., 2004). In support, recurrent gamma oscillation bursts were found for (re)activated information in WM tasks in prefrontal networks (Lundqvist et al., 2016), and the parietal cortex of macaques, when this information was task relevant (Pesaran et al., 2002).

### The theta-gamma code

The theta rhythm (3–8 Hz) is posited to implement a neural coding schema for the organization of multiple items, each item being activated or reactivated at distinct gamma bursts, nested in the theta cycle (Lisman and Jensen, 2013). This concept originates in seminal work on place cells (i.e., neurons that fire at specific spatial location) in the MTL of rodents (O’Keefe and Recce, 1993). Place cells fired at successive theta cycles when a rat navigated through a labyrinth, and the firing of a place cell occurred at earlier phases of the theta rhythm, as the rat proceeded through the labyrinth (a phenomenon named phase precession, see Figure 2B). Specifically, place cells exhibited rhythmic gamma bursts, nested in the ongoing theta cycle in MTL networks (Senior et al., 2008). In this neural coding scheme, the number of gamma bursts, representing single places or items, has been suggested to determine the number of items that can be held in WM (Lisman, 2010), and it has been suggested that the items encoded in MTL networks are linked to neocortical representations. This way, the MTL theta-gamma code may implement a buffer to represent multiple items in a segregated and sequential fashion (Jensen and Lisman, 2005; Lisman and Jensen, 2013; Buzsáki and Tingley, 2018).

Complementing its proposed role as an organizing scheme, theta-gamma coupling has been characterized as a mechanism for attentional selection (Fiebelkorn and Kastner, 2019), with critical evidence from intracranial recordings in humans (Helfrich et al., 2018), and memory formation, with empirical evidence from MTL recordings in rats (Pavlides et al., 1988; Tort et al., 2009). Based on the original finding that place cells are activated before a rat passes specific locations in a labyrinth (O’Keefe and Recce, 1993), we would further like to emphasize that the theta-gamma scheme may implement a predictive code for behavioral navigation (for recent model of rat and human navigation as predictive maps, see De Cothi et al., 2022).

Key to our understanding of the specific neural network properties that may give rise to theta-gamma coupling dynamics (e.g., based on specific properties of cortical pyramidal cells; Lundqvist et al., 2011; Hummos and Nair, 2017) and how these dynamics may implement complex cognitive functions in hippocampal networks (Ursino et al., 2022), are computational models (see Chadwick et al., 2015, for a sequencing model based on fewer assumptions). So called attractor network models have also contributed to the alternative perspective that theta-gamma dynamics may arise as an epiphenomenon of the specific neurophysiology of neural networks, putting into debate their mechanistic function (e.g., Lundqvist et al., 2010; Kovach et al., 2018; Sheremet et al., 2019), discussed in more detail below.

### Alpha oscillations

The most prominent marker of attention processes in the human EEG is the alpha rhythm (8–14 Hz), often functionally merged with the adjacent beta rhythm (14–20 Hz). Berger (1934) first described pronounced alpha activity when his participants had their eyes closed and a sharp decrease in alpha synchronization when participants opened their eyes. By now, it is well established that alpha desynchronization facilitates attention and memory processes (e.g., Klimesch et al., 1997), with empirical evidence for the visual (e.g., Friese et al., 2013), and the auditory domain (e.g., Obleser et al., 2012). Specifically, alpha oscillations are posited to reflect an attentional gating mechanism, which inhibits task irrelevant cortical processes and facilitates neural processes in task relevant cortical networks, shaping a functional neural architecture (Palva and Palva, 2007; Jensen and Mazaheri, 2010). Neural processing may then be reflected in gamma oscillations (Figure 2C). Neurophysiologically, alpha oscillations may result from a pulsed inhibition *via* GABAergic interneurons, which arise in local networks but are likely to be generated in thalamic networks (see Jensen and Mazaheri, 2010, for a more detailed description of the potential underlying mechanisms).

Empirically, intracranial recordings in patients revealed a decrease in NC alpha, which preceded fast gamma oscillations in MTL networks during memory encoding (Griffiths et al., 2019). This complements former evidence that reduced EEG alpha power facilitated information processes when preceding relevant sensory input (e.g., Romei et al., 2008; Busch et al., 2009; see VanRullen, 2016, for a review; Griffiths et al., 2022). An inverse relation between local field alpha desynchronization and neural firing rates in sensory cortical regions has been found in monkeys (Haegens et al., 2011), and the alpha rhythm has been shown to exert top-down influences in the visual cortex of macaques (Bastos et al., 2015; Richter et al., 2017).

## Rhythms of human attention and memory

**FIGURE 3:**
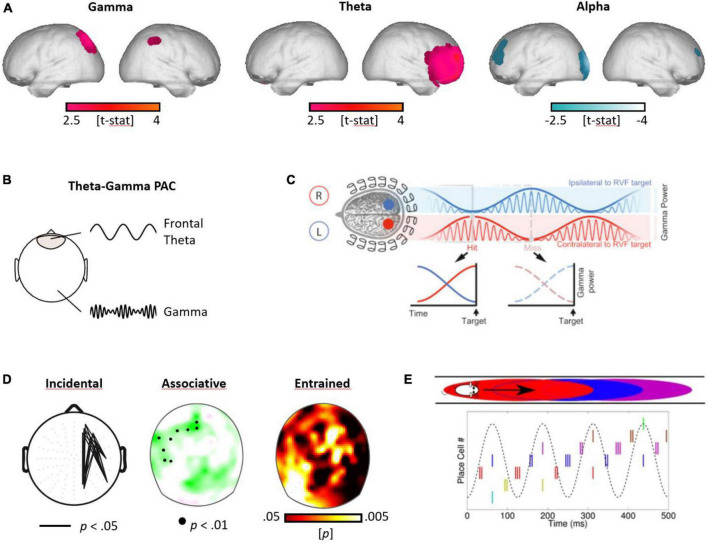
Gamma, theta, and alpha oscillations in human attention, memory, and prediction. **(A)** Gamma, theta, and alpha oscillations during encoding. Subsequently remembered (in contrast to forgotten) items elicited higher parietal gamma, frontal theta, and reduced alpha power in frontal and parietal networks (modified from Friese et al., 2013). **(B)** The concept of phase-to-amplitude coupling (PAC) between the frontal theta rhythm and cortical gamma oscillations. **(C)** Attentional sampling. The modulation of attention, alternating between perceptual target locations in the left and right visual field, with the theta-modulated gamma amplitude predicting the detection of a specific target [cf. Landau et al., 2015; (reproduced with permission)]. **(D)** Memonic updating. Theta-gamma coupling was accentuated for subsequently remembered items in an incidental (Friese et al., 2013) and an associative memory task (Köster et al., 2018), as well as during enhanced encoding for visual perceptual entrainment at the theta rhythm (Köster et al., 2019b). **(E)** Predictive coding. Visualization of the predictive processes during a spatial paradigm, as characterized in mice (O’Keefe and Recce, 1993). The firing of place cells, encoding different locations in a labyrinth, fire at specific phases of the theta rhythm, in a sequential manner, before the mouse enters a specific location (graph from Chadwick et al., 2015; CC4.0).

Here, we propose that neural oscillatory processes may be key to our understanding of the embedded nature of attention and memory processes in the human brain (Cowan, 1997), linking those processes by their shared neural signature. Specifically, based on contemporary theoretical accounts and recent empirical insights, we suggest that gamma, theta, and alpha oscillations implement distinct mechanisms, which act in close concert to shape the functional architecture underlying human attention, memory, and prediction. See Figure 3, for the interplay between gamma, theta, and alpha oscillations during memory formation, as well as the proposed role of the theta-gamma code in attentional sampling, mnemonic updating, and predictive coding.^1^

### Memory items – gamma oscillations reflect the activation and maintenance of neural representations

Perceptual information is promoted along sensory networks, before it converges on sparse representations in conceptual (Quiroga et al., 2005), relational (Collin et al., 2015), and contextual networks (Jacobs et al., 2013). The gamma rhythm is thought to play a decisive role in the activation and maintenance of neural representations in NC and MTL networks (Engel et al., 2001; Herrmann et al., 2004). In the Cowan model, the promotion of sensory signals in primary sensory areas would correspond to the sensory buffer, while the maintenance of neural representations in NC and MTL networks would correspond to activated memory items in LTM.

The role of neural synchronization processes in the gamma range has been well characterized for visual perceptual processes in mammals, from neurons that encode features of the same object in primary visual regions (e.g., Gray et al., 1989), to the synchronization across neural populations (e.g., Fries et al., 2001; Womelsdorf et al., 2007): For example, synchronization processes between neural activity across cell assemblies predicted the performance in a selective attention task (Fries et al., 2001). Extending this research to assess visual cortical processing more broadly, revealed that the promotion of visual signals along the visual hierarchy relies on bottom-up gamma rhythmic activity, feeding neural signals from primary into higher visual cortical regions (e.g., Bastos et al., 2015).

In the wake human brain, gamma oscillations were first identified for object perception processes in the visual cortex using M/EEG (Tallon-Baudry et al., 1996; Tallon-Baudry and Bertrand, 1999), being higher for familiar compared to unfamiliar object stimuli (Tallon-Baudry and Bertrand, 1999). While micro-saccadic eye-movements were shown to elicit physiological artifacts in the gamma frequency range (Yuval-Greenberg et al., 2008), gamma band oscillations for object processing have now been reported after micro-saccadic artifacts are removed (Keren et al., 2010; Hassler et al., 2011; Michalareas et al., 2016; Köster et al., 2018). Besides their role in perceptual processing, gamma oscillations were pronounced in posterior (Sederberg et al., 2003; Osipova et al., 2006; Friese et al., 2013) and MTL regions (Fell et al., 2001; Sederberg et al., 2007) during memory formation and during the maintenance of information in WM (Tallon-Baudry et al., 1998; Kaiser et al., 2003; Daume et al., 2017).

Recent evidence for a functional role of the gamma rhythm in perceptual processes comes from a rhythmic visual entrainment study (Gulbinaite et al., 2019), where perceptual entrainment at the gamma rhythm facilitated, whereas alpha entrainment reduced sensory processes. Another study showed that entrained gamma oscillations, applied *via* transcranial stimulation, facilitated perceptual processes in primary auditory networks (Wöstmann et al., 2018).

Critically, the sampling, segregation and integration of perceptual information requires a neatly timed coordination of neural information processing. This brings us to the core element of our framework, the theta-gamma neural code.

### The central executive – theta-gamma coupling reflects attentional sampling, mnemonic updating and predictive coding

In the Cowan model, the central executive samples, maintains, and elaborates memory items to utilize them for controlled behavior. Based on evidence from cognitive control tasks (e.g., stoop and odd-ball paradigms) the prefrontal theta rhythm has been ascribed a key role in executive control processes (cf. Cavanagh and Frank, 2014). In the concept of the theta-gamma neural code (Lisman and Jensen, 2013), the theta rhythm governs the cyclic selection, maintenance, and integration of memory items in NC and MTL networks, as reflected in gamma bursts nested within the theta cycle in an ordered fashion. Based on the principal function of central executive processes in behavior (Cowan, 1997) and the predictive role of theta-gamma coupling in spatial navigation (O’Keefe and Recce, 1993), we further propose that this information may be utilized to navigate towards future goals. As characterized below, the theta-gamma code may serve the remapping of real time events onto a faster neural time scale (cf. Skaggs et al., 1996; Foster and Wilson, 2007) to solve key problems of neurophysiological computation: attentional sampling, mnemonic updating, and prediction, and thereby implement the embedded nature of attention and memory processes on the neural architecture of the human brain.

In humans, the theta rhythm itself has been found to increase in PFC and MTL networks for memory encoding (Fell et al., 2003; Sederberg et al., 2003; Osipova et al., 2006; Rutishauser et al., 2010; Friese et al., 2013; Köster et al., 2018), as well as WM maintenance (Jensen and Tesche, 2002; Scheeringa et al., 2009; Khader et al., 2010), which has been specifically associated with the rehearsal of the encoded information (Fuentemilla et al., 2010). PFC and MTL networks have been shown to interact during mnemonic processing at a theta pace (Axmacher et al., 2008; Staudigl and Hanslmayr, 2013; Kaplan et al., 2014; Backus et al., 2016). Supporting its role as an explicit control mechanism in memory formation, theta power has been accentuated for the encoding in explicit (Köster et al., 2017b) and volitional encoding conditions (Estefan et al., 2021).

The functional role of the theta rhythm for memory formation processes has recently been substantiated by rhythmic perceptual entrainment studies. These studies demonstrate that the perceptual entrainment of theta oscillations enhances memory within the visual domain (Köster et al., 2019b), and across the visual and auditory domain (Clouter et al., 2017; Albouy et al., 2022).

Theta-gamma coupling in the human brain has been found during prefrontal control processes (Canolty et al., 2006), WM maintenance (Mormann et al., 2005; Daume et al., 2017) and memory formation (Friese et al., 2013; Heusser et al., 2016; Köster et al., 2018) in NC and MTL networks. First evidence that the theta-gamma code reflects the serial organization of distinct memory items in the human brain, comes from intracranial recordings (Bahramisharif et al., 2018). In this study, the gamma bursts of letter sensitive neurons were temporally coupled to the theta phase, in the order of the letters maintained during a Sternberg WM task. Besides its involvement in cognitive control and memory processes, there is accumulating evidence that the theta rhythm guides the sampling of visual perceptual information by rhythmically facilitating perceptual processes in the gamma rhythm (Landau et al., 2015; Helfrich et al., 2018; Peters et al., 2018; Re et al., 2019). For example, this has been shown for theta-gamma PAC processes in primary sensory regions, where the gamma amplitude fluctuated at the theta rhythm and predicted the detection of a target (Landau et al., 2015).

Within the theoretical model proposed by Cowan (1997), we hypothesize that the prefrontal theta rhythm is the pacemaker of central executive processes, acting on neural representations (memory items), as reflected in the theta-gamma neural code. Specifically, the theta-gamma code may represent several bits of information in an organized and time-compressed manner to solve key problems of neural computation: attentional sampling, mnemonic updating, and behavioral prediction. In the following, we will further elaborate on these three proposed functions of the theta-gamma neural code.

#### Attentional sampling

Distributed sensory processes need to be integrated (Engel and Singer, 2001) and, at the same time, segregated as distinct perceptual units. This could be realized by the promotion of perceptual information along the visual hierarchy at a theta pace (Fries, 2015) and the cyclic reactivation of item information at distinct gamma bursts, nested in the theta phase (Jensen and Lisman, 2005; Lisman and Jensen, 2013). Such a mechanism may serve the selective attention to novel information and the simultaneous maintenance of attended items in WM.

In the human brain, gamma activity has been found to fluctuate at the theta rhythm in primary visual areas corresponding to potential target locations in the left versus right hemifield, facilitating the detection of a target (Landau et al., 2015; Figure 3C). This study was inspired by findings in the macaque visual cortex, showing that the gamma coherence between V1 and V4 was reset at a theta pace (Bosman et al., 2012), and that theta and gamma oscillations serve the bottom-up promotion of sensory signals along the visual hierarchy (Bastos et al., 2015). Critically, the sampling of visual information (i.e., overt attention) by saccades and micro-saccades likewise show a periodicity of around 5 Hz in humans (Otero-Millan et al., 2008), and in the macaque visual cortex, the theta rhythm has been shown to be linked to microsaccadic eye-movements (Bosman et al., 2009). A reset in visual networks at the theta rhythm through (micro)saccades may potentially serve the segregation of different bits of perceptual information (cf. Fries, 2015).

Linking frontal control processes to eye-movements, in macaques, the theta rhythm in the FEF has been shown to modulate the gamma activity in the lateral intraparietal area (LIP), as part of the attentional network, which enhanced perceptual sensitivity (Fiebelkorn et al., 2018). The FEF exhibits strong connections with executive networks in the PFC and the superior colliculi (Hanes and Wurtz, 2001) and may thus serve as a hub between PFC control processes and periodic attentional sampling mechanisms of ocular motor system, to establishing a visual perceptual sampling mechanism at the theta pace.

#### Mnemonic updating

To maintain a coherent and up-to-date internal map of time and space, perceptual information must be integrated and updated continuously. Hebbian learning, the principle that cells that fire together, wire together (Hebb, 1949) requires the neatly timed activation within and across cell assemblies (see Fell and Axmacher, 2011, for a review of neural oscillatory mechanisms that facilitate neural plasticity). Specifically, perceptual information that occurs at temporally distant points in real time has to be (re)activated at a faster pace, with close temporal proximity, at the neural level, to enable long-term potentiation (LTP) processes in MTL networks, with tetanic inputs at the pre- and post synapse being a prerequisite for the formation of synaptic connections, a mechanism well characterized at the cellular level (see Bear and Malenka, 1994, for a review).

Regarding the theta-gamma code in memory formation, the precisely timed neural input of gamma bursts to the down phase of the theta rhythm has been shown to facilitate LTP processes in the MTL of rodents (Pavlides et al., 1988) and that theta-gamma coupling was increased for novel encoded item-context associations in the rat MTL (Tort et al., 2009). By now, there is abundant evidence for a key role of the theta-gamma PAC in human memory formation (Friese et al., 2013; Heusser et al., 2016; Köster et al., 2018, 2019b; see Figure 3D). For example, in one of our studies, we found an increase in frontal theta-gamma coupling, specific for the encoding of novel item-color associations (Köster et al., 2018; see also Griffiths et al., 2021). These findings are complemented by intracranial recordings, where the maintenance of specific letters in a Sternberg task led to gamma bursts of letter sensitive cells, nested and ordered within the ongoing theta local field potentials (Bahramisharif et al., 2018).

When manipulating memory encoding by rhythmic visual entrainment, successful encoding was found to depend on a theta-gamma coupling pattern elicited in a theta entrainment condition, rather than by the theta power *per se* (Köster et al., 2019b). In another study, WM capacity could be increased experimentally, when the theta rhythm was slowed down by transcranial alternating stimulation (Vosskuhl et al., 2015), which supported the hypothesis of the authors that with prolonged theta cycles, an increased number of items could be rehearsed and therefore maintained within each cycle. Further experimental evidence for the involvement of theta-gamma coupling processes in WM comes from a study by Alekseichuk et al. (2016), who enhanced WM performance by theta-gamma stimulation protocols, and empirical findings that WM capacities can be revived by a theta-gamma stimulation applied in elder participants (Reinhart and Nguyen, 2019).

Given its central role in human memory formation, theta-gamma coupling is proposed here to serve as a temporal coding scheme for the continuous integration of perceptual information, in order to update and retain a coherent representation of time and space. Specifically, the theta-gamma code may compress real time events onto a faster neural timescale to facilitate the formation of novel associations in MTL networks through the facilitation of LTP mechanisms. Such a continuous update of internal representations is essential for a real-time internal map of the outer world, but also to guide experience-based predictions and behavior.

#### Predictive coding

The prediction of future events in the environment and the effects of behavioral navigation on changes in perceptual input is integral to human cognition (O’Regan and Noë, 2001; Friston, 2010; Engel et al., 2013). Computationally, translating sensory information into experience-based predictions, requires associative processes to speed up and advance ahead of real time, to form predictive models (i.e., in the sense of active inference; Friston, 2010) that guide behavioral navigation.

We propose here that the theta-gamma neural code is an ideal candidate to implement such a predictive code. This concept is inspired by the original observation that place cells in the MTL of rats are activated prior to the actual location, which is represented by the specific place cell (O’Keefe and Recce, 1993; see Figure 3E). Associative networks in the MTL circuit may generate context-dependent predictions, based on given sensory inputs. That is, based on its present location in the labyrinth, the rat predicts its behavioral outcomes, by activating spatial representations ahead of real time, or, in other words, the rat forms a predictive model that guides behavioral navigation through the labyrinth.

First evidence from human MTL recordings suggests an involvement of the theta rhythm in organizing different representations during behavioral navigation (Kunz et al., 2019) and memory based behavioral responses (Estefan et al., 2021; Ter Wal et al., 2021). For example, in a study by Estefan et al. (2021), the MTL showed increased theta power and theta-gamma coupling for volitional control in a spatial memory task. Critically, theta activity predicted the reactivation of a context-specific memory content in MTL networks and the theta-phase was opposite for the encoding and the reactivation of stimulus specific information. This finding is consistent with the idea that distinct phases of the theta rhythm serve the sampling and encoding of novel information and the retrieval of former experience, based on this information (Hasselmo et al., 2002; Hasselmo and Eichenbaum, 2005), which may be utilized for behavioral navigation (for further evidence, see Rizzuto et al., 2006; Kerrén et al., 2018; for first evidence that retrieval processes are also associated with theta-gamma coupling, see Köster et al., 2014).

Importantly, attentional sampling itself can be conceptualized as a predictive process, namely, where relevant information in the environment may be found based on current perceptual inputs, which may be characterized as an attentional mnemonic sampling loop that governs cognitive foraging. Closing the loop, it has been well described that the prefrontal theta rhythm in the ACC indexes the processing of prediction errors (see Cavanagh and Frank, 2014 for a review), which may promote the integration of novel, unexpected events, as they occur, to update and refine predictive models in real time.

### Activated long-term memory – the alpha rhythm gates information processing in semantic networks

The desynchronized alpha rhythm is proposed to reflect a gating mechanism, shaping a functional neural architecture involved in explicit and implicit processes alike (Palva and Palva, 2007; Jensen and Mazaheri, 2010). In the Cowan model, this function of the alpha rhythm corresponds well to the concept of activated information in LTM (Cowan, 1988; indexed by the gray cloud in Figure 1A). Activated neural representations may be involved in explicit mnemonic processes, underlying cognitive control, or in implicit processes, involved in automated neural processing and routined behaviors, not requiring mental elaboration or mnemonic updating (Parkin et al., 1990).

Contrasting the attentional mechanisms proposed for the increased theta rhythm (see above) versus the desynchronization in the alpha band, the concept of attentional sampling at the theta pace corresponds closely to the idea of selective attention processes, while the alpha rhythm may reflect sustained attention processes (Raz and Buhle, 2006; Posner and Rothbart, 2007). In Cowan’s (1988) model the central executive operates on a subset of activated LTM information. Accordingly, theta-gamma coupling mechanism may operate on a subset of the overall activated neural networks, potentially reflected by decreased alpha activity.

Noteworthy, Klimesch (2012) suggested a very similar concept of the desynchronized alpha rhythm, proposing that it reflects the interpretation of sensory inputs based on a semantic “knowledge system.” Likewise, Klimesch separated attentional processes in the theta rhythm (indexing the explicit processing of novel perceptual information) from attentional mechanisms reflected in the alpha rhythm (mainly involved in suppressing task irrelevant neural processes; see also Klimesch, 2013). This suggests a less specific cross-frequency interaction between alpha and gamma oscillations during mnemonic processes, or, as Jensen and Mazaheri (2010) have characterized it, a “positive correlation between gamma power in task-relevant regions and alpha power in task-irrelevant regions,” with no specific PAC pattern (for a similar proposal for WM processes, see also Roux and Uhlhaas, 2014).

Consistent with the idea that theta and gamma oscillatory processes act on functional networks shaped by alpha based gating mechanisms, desynchronized alpha power in parietal and frontal networks has often been found to coincide with increased theta power during memory formation (Klimesch et al., 1997; Mölle et al., 2002; Osipova et al., 2006; Friese et al., 2013; Köster et al., 2018), as well as increased gamma power (Osipova et al., 2006; Friese et al., 2013; Köster et al., 2018). Conversely, alpha oscillations are commonly found to be accentuated over parietal regions during a WM task (Jensen and Tesche, 2002; Scheeringa et al., 2009; Khader et al., 2010), which may serve the inhibition of interfering sensory information during WM maintenance (Cooper et al., 2003).

Support for the causal relevance of alpha oscillations in gating information processes in human primary sensory networks comes from two recent transcranial stimulation studies. Here, the experimental application of the alpha rhythm led to reduced gamma oscillations and impaired the detection of a visual stimulus, when applied to the visual cortex (Herring et al., 2019), and reduced auditory discrimination performance, when applied over primary auditory regions (Wöstmann et al., 2018).

**FIGURE 4:**
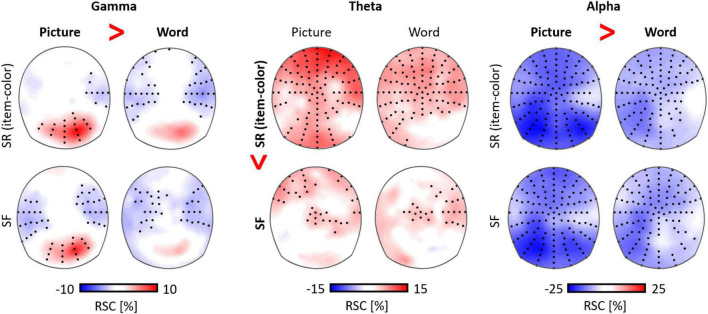
Distinct functions of gamma, theta, and alpha oscillations in visual information processing (pictures versus words) and memory formation (item-color associations; Köster et al., 2018). Theta power was higher for subsequently remembered (SR) versus subsequently forgotten (SF) item-color associations, while parietal alpha suppression and gamma power were higher for pictures compared to words, but did not differ between encoding conditions (RSC: relative signal change).

Regarding the functional separation between the theta and the alpha rhythm, the encoding of semantic information has been associated with alpha suppression, but the explicit encoding of perceptual features was associated with high theta power (Hanslmayr et al., 2009). Similarly, an explicit encoding instruction led to pronounced theta activity, but alpha power did not differ between an implicit and an explicit encoding condition across diverse age groups (Köster et al., 2017b). In this study, alpha suppression showed a close correspondence with age differences in the response times in a semantic encoding task. In a recent study, we directly separated mnemonic from perceptual processes, by contrasting the encoding activity for pictures versus words (Figure 4; Köster et al., 2018), which had to be associated with a specific background color. Alpha suppression was higher for stimuli with rich perceptual features (i.e., pictures), compared to low perceptual features (i.e., words), but did not differ between encoding conditions (subsequently remembered versus forgotten). However, in this task the theta rhythm and theta-gamma coupling processes predicted successful associative memory encoding. Finally, Williams et al. (2019) contrasted analytic cognitive processing (simple number arithmetic) with highly automated semantic processing (repeating a number) and found increased theta power in the analytic but reduced alpha power in the semantic condition.

### Summary – brain rhythms orchestrate an attentional mnemonic updating loop

When awake, central executive processes (in the PFC, along with attention and mnemonic control networks) continuously sample information from the environment (attentional selection). Novel information is interpreted based on semantic knowledge (LTM items in NC networks) and integrated, to retain a coherent representation of time and space (in fast updating MTL networks). This up-to-date mental map allows for behavioral navigation in the light of current goals (monitored in PFC networks).

**FIGURE 5:**
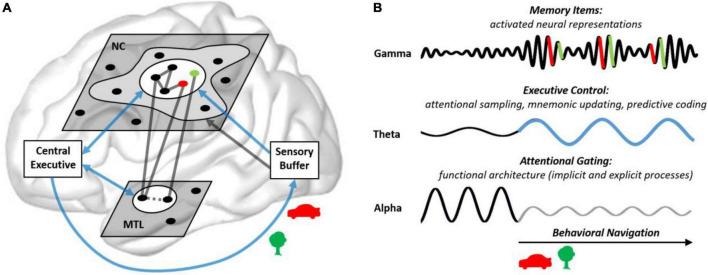
How brain rhythms may implement the embedded nature of human attention and memory. **(A)** The central executive (PFC in coalition with attentional and mnemonic control networks, see Figure 1B) sets the focus of attention on a subset of memory items (indicated by the white circles; see Figure 1A) in the NC (semantic information) and the MTL (contextual information). For example, on novel items which are attended visually and integrated into associative networks, indexed by the green and red dots (e.g., representing a red car and a green tree). In MTL networks, novel items are integrated (dotted line) into a spatio-temporal map of the outer world. **(B)** Activated memory items, in distributed cell assemblies, synchronize their firing in gamma bursts (>30 Hz). The central executive (blue arrows) selects and integrates memory items and utilizes them for predictions at a theta pace (3–8 Hz). Alpha oscillations (8–14 Hz) gate information processing in semantic networks by inhibiting task irrelevant networks. This shapes a functional architecture underlying explicit and implicit mnemonic processes (including automated processes, not requiring the central executive; gray cloud in **A**). An integral aspect of the embedded process model is that explicit as well as implicit mnemonic processes ultimately serve behavioral navigation (including visual foraging).

Here we propose that neural rhythms reflect the integrated nature of attention and memory in the human brain, by implementing key elements of the embedded-process model (see Figure 5 and Table 1). In this framework, the prefrontal theta rhythm is the pacemaker of the central executive, which activates different bits of information in NC and MTL networks (memory items), reflected in gamma bursts, forming the theta-gamma neural code. Specifically, the theta-gamma code may represent multiple memory items in a sequential and time-compressed manner, to solve key problems of neural computation: First, the sampling of novel bits of information from the environment and the (re)activation of memory items, as segregated information in a sequential way. Second, the temporal compression of real time events facilitates LTP processes in MTL networks, which are crucial for the continuous updating of internal representations, here coined mnemonic updating. Third, speeding up neural processing ahead of real time enables prediction, the simulation of events in the environment and the outcomes of behavioral navigation, based on current information and former experience. It should be noted that, in our framework, we conceive the sampling of information by selective attention a prediction-based behavior in itself, because it reflects the anticipation where relevant information may be found.

**TABLE 1:** Overview of the proposed framework.

Brain rhythm	Psychological function	Key references	Counterpart in embedded process model (Cowan, 2016)	Implementation on brain structures
Gamma	Promotion and binding of sensory information, (re)activated neural representations	Engel and Singer, 2001; Fries, 2015	Sensory buffer and activated items in LTM	Information processing in sensory areas, (re)activated representations in the NC and context information in the MTL
Theta	Pacemaker of human cognitive control and mnemonic processes, by temporal remapping	Lisman and Jensen, 2013; Cavanagh and Frank, 2014	Central executive (setting the focus of attention)	PFC networks and attentional and mnemonic control networks orchestrate human cognitive control (activated representations in NC and MTL networks → theta-gamma PAC)
Theta-gamma PAC	Attentional sampling	Lisman, 2010; Fries, 2015; VanRullen, 2016; Fiebelkorn and Kastner, 2019	Attended sensory information and (re)activated items in LTM	Visual sensory information processing (e.g., by steering eye-movements *via* the FEF and the superior colliculi), (re)activation of NC and MTL representations
	Mnemonic updating	Fell and Axmacher, 2011; Roux and Uhlhaas, 2014; Hanslmayr et al., 2016	Formation of novel associations between activated items	LTM formation in the MTL serves the continuous integration of novel information
	Prediction and generation of action plans	O’Keefe and Recce, 1993	Guidance of controlled behavior	Utilization of activated representations in NC and MTL networks to form predictive models, steered by the PFC
Alpha	Attentional gating	Jensen and Mazaheri, 2010	Gating of information in the sensory buffer and LTM	Facilitation (suppressed alpha) and inhibition (increased alpha) of neural information processing
Alpha-gamma association	Functional architecture of attention and memory processes (explicit, implicit)	Klimesch, 2013; Roux and Uhlhaas, 2014	Activated items in LTM involved in explicit (attended) and implicit (habituated) processing	Activation of task relevant PFC, NC and MTL networks, by the deactivation of irrelevant processes

Brain rhythms, their psychological function, key references, corresponding elements in the embedded-process model (Cowan, 2019), and their proposed implementation in the human brain.

In summary, the theta-gamma code implements an attentional mnemonic updating loop that samples, updates, and utilizes perceptual information to form predictions and guide behavior: The PFC initiates a neatly ordered processing sequence, sampling and integrating novel information, bit by bit, at a theta pace (e.g., by steering eye-movements *via* the frontal eye-field and the superior colliculi). This information is interpreted in the light of semantic associative networks (in the NC), the representation of time and space (in the MTL) and current goals (encoded in PFC and ACC networks), to evaluate and integrate perceptual information as they occur in real time, and to guide future behavior.

Critically, we propose distinct roles for the theta and the alpha rhythm. The theta-gamma code reflects explicit control processes, which steer the (explicit) dialog between PFC, MTL and NC networks. In contrast, the alpha rhythm reflects the activation of associative, semantic networks involved in explicit but also in implicit processes (the LTM storage). The alpha rhythm may also gate the implicit processing of environmental information that underlies automated behavior, namely information processing that is highly habitualized and does not rely on explicit mental elaboration (cf. Cowan, 1997).

## Future perspectives

The main purpose of our framework is a conceptual link between psychology and cognitive neuroscience, toward a mutually informed theory of human attention and memory. Focusing on oscillatory dynamics, we close the conceptual divide between psychology and brain mapping approaches by specifying how neural rhythms may implement the core elements of the embedded-process model of attention and memory on the neural architecture of the human brain (Cowan, 1997).

The past decades have seen essential advances in our functional understanding of neural rhythms in mnemonic processes. This includes the promotion of sensory signals in the visual system (Engel et al., 2001; Varela et al., 2001; Fries, 2015), the theta rhythm in cognitive control (Cavanagh and Frank, 2014), the theta-gamma coding schema in neural organization (Lisman and Jensen, 2013), WM (Lisman, 2010; Roux and Uhlhaas, 2014), and LTM processes (Hanslmayr et al., 2016), as well as the role of the theta and alpha rhythm in attentional sampling (VanRullen, 2016; Fiebelkorn and Kastner, 2019) and gating (Jensen and Mazaheri, 2010; Klimesch, 2012). Our framework aims at integrating those distinct perspectives on human attention and memory, based on their shared neural signatures. While the neurophysiological mechanisms are well understood and have been specified for some elements of this framework, down to the cellular level (e.g., LTP process in the MTL of rodents; Bear and Malenka, 1994), they remain less specific for other elements of this framework (e.g., explicit and implicit memory processes being reflected by theta-gamma versus alpha pattern, respectively). Thus, our framework can only be understood as a starting point for an in-depth examination of how attention and memory processes are realized and interlinked with each other neurophysiologically. Likewise, by providing principal ideas on how different aspects of human cognitive functions may be interlinked, we hope to inform computational models, to test specific hypotheses that can be derived from our framework (see below).

In this tenant, with our framework we hope to inspire the research and theory in psychology and cognitive neuroscience, and the integration of these perspectives (for the contemporary debate in psychology, see also Norris, 2017; Cowan, 2019). We are convinced that a deeper and integrated understanding of oscillatory brain processes will certainly further advance our conceptual understanding of human attention and memory, and their embedded nature, building the bedrock of human cognitive functions. In the following, we highlight some exciting and currently emerging avenues for future research and how these may benefit from the embedded process view proposed here.

### How do brain rhythms coordinate attention and memory?

A central element of our framework is the theta-gamma code in human cognitive control (i.e., central executive processes). Namely, the theta-gamma code is proposed to remap real time events onto a faster neural timescale to solve key problems of neural computation: attentional sampling, mnemonic updating, and prediction.

While initial evidence exists for each of these specific processes in isolation, the concept of their embedded nature requires the orchestration of these processes across human brain networks in shaping the functional architecture underlying human cognitive functioning. Yet, it is not well understood how neural rhythms are ultimately coordinated and interplay with each other across brain regions and distinct cognitive processes. For example, the theta-gamma code is ascribed the role of a PFC and MTL control mechanism (Buzsáki, 1996; Anderson et al., 2010), acting on perceptual information in a top-down manner. On the contrary, there is recent evidence for a potential bottom-up function of the theta-gamma code in the visual cortical networks (Landau et al., 2015; Lowet et al., 2016), including the finding that visually entrained theta dynamics facilitated encoding, by driving theta-gamma coupling processes (Köster et al., 2019b). A specific proposal of our framework, which may resolve this controversy, is that control processes in the PFC and MTL act in concert with the (oculo)motor and the visual system, to implement attentional sampling. This way, novel perceptual information in sensory networks, reflected by bottom-up processes in the theta and gamma band (Bastos et al., 2015), may be reset and sampled, bit by bit, by frontally guided control processes at a theta pace (Fries, 2015; VanRullen, 2016).

In fact, the attentional mnemonic updating loop characterized here, allows for specific hypotheses on how the theta-gamma coding schema may guide experience based visual exploration and the integration of novel visual information. For example, by combining the assessment of gaze behavior (using eye-tracking) and neurophysiological data (M/EEG), it would be intriguing to further scrutinize the interplay between the prefrontal theta phase, eye-movements and gamma bursts in perceptual and semantic networks. That is, how the theta rhythm in PFC and MTL networks may be linked to the guidance of (micro-)saccades and visual cortical processes and inform subsequent oculomotor processes.

Another outstanding query on the coordination of oscillatory processes regards the co-occurrence and interplay between increased theta and reduced alpha power. One potential explanation may be a shift (tilt) in the operating rhythm, from alpha to theta, in brain regions involved in central executive processes (cf. Hanslmayr et al., 2016). This is, the activity may switch from an idle state, the alpha rhythm, to a lower working pace, the theta rhythm. Alpha desynchronization may then be epiphenomenal to high alpha power in the idle state (i.e., the default mode; Raichle, 2015). However, our framework makes more specific predictions, namely alpha desynchronization being a marker of implicit and explicit mnemonic processes alike, but theta and theta-gamma coupling being a marker of explicit memory processes. This conception calls for a more fine-grained functional dissociation of the neural computations reflected in alpha versus theta(-gamma) oscillatory signatures.

In our framework, we emphasize that the ultimate purpose of information processing is behavioral navigation. However, beyond the control of eye-movements, we do not specify how do attention and memory control processes may steer behavioral responses on the level of dynamic recruitment of neural processes in the motor system. There is good evidence that the beta rhythm (15–30 Hz) is implicated in the control of the motor system (for reviews, see Engel and Fries, 2010; Jenkinson and Brown, 2011; Khanna and Carmena, 2015). We are not aware if motor control processes have formerly been linked, theoretically or empirically, to the oscillatory dynamics of human cognitive control characterized here. The integration of cognitive and motor control processes at the level of temporally resolved neural dynamics, is a major challenge for future research (cf. Waschke et al., 2021), which may benefit from a broader conceptual framework of human attention and memory suggested here.

### Modeling neural network dynamics

A critical tool to test and further specify the assumptions on neural oscillations and their functional relevance are computational models, which allow to specify and test neurophysiological assumptions in detail. In the following, we will exemplify critical contributions that have been made to the conception of the theta-gamma code and highlight potential future directions.

A computational model of how the theta-gamma code may support the restoration and sequence coding of several learned items in WM, has recently been developed based on hippocampal connectivity and Hebbian learning principles (Ursino et al., 2022). This model provides specific support for the idea that the theta-gamma code indeed supports those functions, derived from specific network characteristics. Developing their model, the authors remarked that a dissociation of the storage of external information in WM can hardly be dissociated from more generalized (semantic) learning phenomena. Namely, the model also restored related items in the absence of external input in or out of their original sequence. Just like in the conceptual model proposed here the authors argued for an integrated view on those memory processes.

At the same time, computational models have provided a crucial alternative view (e.g., Lundqvist et al., 2010, 2011; Herman et al., 2013), namely that critical aspects of the theta-gamma code can arise in attractor network models, through information processing dynamics in models building on detailed neural and synaptic connectivity assumptions. For example, the theta rhythmic pattern has been found to arise from intrinsic network activity in spiking (Hummos and Nair, 2017), but also in non-spiking networks (Lansner et al., 2013), and phase amplitude coupling has been interpreted as a high-order spectrum estimation (Kovach et al., 2018), which may reflect the cross-frequency coupling feature of any non-linear dynamic system (Sheremet et al., 2019). Yet in other simulations, it was found to be beneficial for working memory operations if a significant part of the excitatory current from distant part of an active cell assembly arrives out of phase with the local gamma oscillations (Lundqvist et al., 2010). In this context, higher frequencies may not be assigned an important functional role as a pacemaker.

We hope that the perspective taken here may inform computational models, for example, by providing a concept of how WM and LTM processes may interact. In turn, attractor models may be crucial for our understanding how neural oscillatory dynamics characterized in our framework may arise from the self-organized nature of the networks of the human brain.

### Understanding how brain rhythms guide and change over human development

Infancy and childhood are periods of intense brain development and learning, where developing humans built up basic representations of the world around them (cf. Köster et al., 2020). It is therefore a major challenge to better understand the ontogenetic fundamentals of neural oscillatory dynamics and their involvement in brain development. The central questions are how the mature associative network structure of the adult brain develops, how neural oscillatory dynamics contribute to the major developmental changes in the neural architecture, and how oscillatory dynamics change over time? In fact, these scientific inquiries are closely interlinked: neural oscillatory dynamics may lead to developmental changes. At the same time, the development of brain structures may lead to developmental changes in its operating rhythms (Köster et al., 2017b; Cellier et al., 2021).

The theta rhythm is operative from very early in development, in the processing of novel and unexpected information (Köster et al., 2019a,^2021^) and supports the brain in building up basic representations of the environment (Xie et al., 2021). This is, basic category representations have been found to be based on theta activity in infancy, when those representations are still building up, and then rely on alpha activity in the mature adult brain, reflecting a faster and more efficient activation of those categories, likely based on semantic processes (Xie et al., 2021). There is accumulating evidence for a developmental shift in the dominant locus of the theta rhythm, from parietal to prefrontal networks, and a maturational change in the alpha rhythm (Köster et al., 2017b; Cellier et al., 2021). The parietal theta rhythm may index early learning processes in semantic networks and those networks may become more efficient and allow the implicit semantic processing, reflected in alpha dynamics, as the brain matures throughout childhood (Köster et al., 2017b). This fits well with theoretical accounts on memory development (Ofen and Shing, 2013), presuming that children are still building up semantic network structures and base their semantic judgments on increasingly differentiated associative networks.

The framework introduced here may help us to further specify our assumptions about human brain development, at a verbal and preverbal age (for a developmental account of the Cowan model, see also Cowan, 2016). For example, given the major role ascribed to theta-gamma coupling processes in building up basic representations, a crucial step into this direction is to make gamma oscillations accessible in the EEG of young infants (cf. Köster, 2016).

### Understanding the nature of human memory representations

Another intriguing question regards the physiological nature of neural representations (for recent developments in the field, see Deuker et al., 2016; Bellmund et al., 2018) and how they change over time, in the process of mnemonic updating and consolidation (Nadel and Moscovitch, 1997). This is, what is the specific information encoded in neural networks in the MTL, mainly holding intermediate, contextual information, and the NC, as a LTM storage for specific and general item information (Lisman and Jensen, 2013), and how do these representational networks interact and change in the course of the consolidation process. We know that sleep plays a primary role in memory consolidation (Marshall and Born, 2007; Diekelmann and Born, 2010). During sleep newly acquired associations from context dependent MTL networks may be transferred into slowly adapting semantic networks (Deuker et al., 2013), with critical effects on memory representations (Köster et al., 2017a). However, neural representation may also change in the wake human brain, in particular after repeated exposure to sensory information (e.g., Bäuml and Trißl, 2022). The formation of novel representations may initially rely on explicit control processes, reflected in the theta-gamma code, before they become gradually integrated into semantic networks, reflected in changes in the alpha rhythm (Graetz et al., 2019). For future research, it would be intriguing to further track the changes of neural representations in the MTL and NC over time, for example, across repeated presentations or across episodes of sleep. Combining neural oscillatory analyses, proposed as the key carriers of this information in associative networks in the current framework, with the tools from decoding and representational similarity analyses provide exciting potentials for such advances, which have rarely been exploited.

### Variations in brain rhythms as a pathway to an individualized neuroscience

Regarding the primary role of the theta rhythm in cognitive control, there is also opposing evidence, indicating reduced theta activity during successful encoding (Burke et al., 2013; Greenberg et al., 2015; Griffiths et al., 2016). While these diverging results may be due to specificities of the applied tasks (and the afforded processing dynamics), they may also result from inter-individual variations in the theta rhythm (Klimesch et al., 2001). As a critical hint into this direction, in our studies we adjusted frequency bands individually, to the peak theta and alpha frequencies, and found that the prefrontal theta rhythm predicted subsequent memory performance with high consistency (Friese et al., 2013; Köster et al., 2018, 2019b). This builds on the principal insight that frequency spectra vary profoundly between individuals (Klimesch, 1999), which is hardly accounted for in empirical studies. In future, an advanced understanding of inter-individual differences in the operating rhythms of the brain, in particular the theta rhythm, and their relation to inter-individual differences in mnemonic functions, can pave the way toward an individualized neuroscience. An integrated and global conception of what the gamma, theta, and alpha rhythms entail, as introduced in the present framework, may support the targeted investigation of individualized frequencies and their application in neuromodulation approaches.

### Employing our functional understanding of brain rhythms for neuromodulation

A major recent development is the targeted experimental manipulation of neural rhythms in the wake human brain (Hanslmayr et al., 2019), first, to experimentally test their functional relevance in cognitive processes and, second, for applying our understanding from human functioning in neuromodulation and neuroenhancement (Alekseichuk et al., 2016; Clouter et al., 2017; Köster et al., 2019b; Reinhart and Nguyen, 2019). These approaches may benefit from individualized frequency adjustments or from locking neural entrainment to ongoing neural dynamics. That is, one could link the pace and the phase of the stimulation techniques to ongoing theta processes based on online electrophysiological monitoring. Our deepening functional understanding on human brain rhythms may unfold entirely new potentials to modulate cognitive functions in the wake human brain. In future, an integrated understanding of the embedded nature of human attention and memory may form an essential basis for clinical applications, neural enhancement, and therapy.

## Author contributions

MK wrote the manuscript. MK and TG revised the manuscript. Both authors approved the submitted version.

## Footnotes

1 Note that in this article we focus on the visual modality. However, as highlighted at some points in the manuscript, there are good reasons to assume that other modalities rely on very similar mechanisms, such as, for example, the auditory modality (e.g., Giraud and Poeppel, 2012).
